# Harris' Dictionary of Dental Surgery

**Published:** 1867-10

**Authors:** 


### BIBLIOGRAPHICAL NOTICE.

*Harris' Dictionary of Dental Surgery. Third Edition, Revised and Enlarged, by Ferdinand J. S. Gorgas. M.D., D.D.S., Professor of Dental Surgery in the Baltimore College.*

We have received from those well known publishers of Medical, Dental and Scientific books, Messrs Lindsay and Blakiston of Philadelphia, a copy of this work, the mechanical execution of which far excels that of the former editions. By subjecting the work to a careful revision, the editor has endeavored to bring it up to the present state of the Science, and make it worthy the attention of the profession. About three thousand new terms have been added, and additions and corrections made to the definitions of many others.

Among the great number of important additions are the doses of the more prominent medicinal agents; a table of the muscles arranged according to their actions; and the different classes of poisons with their antidotes. Many of the old formulæ, which are now obsolete, have been omitted, together with descriptions of the treatment of diseases of the dental organs, where the same appears in the author's Principles and Practice of Dental Surgery.

